# Critical Role of Aquaporins in Cancer: Focus on Hematological Malignancies

**DOI:** 10.3390/cancers14174182

**Published:** 2022-08-29

**Authors:** Alessandro Allegra, Nicola Cicero, Giuseppe Mirabile, Gabriella Cancemi, Alessandro Tonacci, Caterina Musolino, Sebastiano Gangemi

**Affiliations:** 1 Division of Hematology, Department of Human Pathology in Adulthood and Childhood “Gaetano Barresi”, University of Messina, 98100 Messina, Italy; 2 Department of Biomedical, Dental, Morphological and Functional Imaging Sciences (BIOMORF), University of Messina, 98100 Messina, Italy; 3 Clinical Physiology Institute, National Research Council of Italy (IFC-CNR), 56124 Pisa, Italy; 4 Allergy and Clinical Immunology Unit, Department of Clinical and Experimental Medicine, University of Messina, 98100 Messina, Italy

**Keywords:** aquaporins, water permeability, cancer, leukemia, lymphoma, myeloma, prognosis, biomarker, angiogenesis, apoptosis

## Abstract

**Simple Summary:**

Aquaporins are proteins able to regulate the transfer of water and other small substances such as ions, glycerol, urea, and hydrogen peroxide across cellular membranes. AQPs provide for a huge variety of physiological phenomena; their alteration provokes several types of pathologies including cancer and hematological malignancies. Our review presents data revealing the possibility of employing aquaporins as biomarkers in patients with hematological malignancies and evaluates the possibility that interfering with the expression of aquaporins could represent an effective treatment for hematological malignancies.

**Abstract:**

Aquaporins are transmembrane molecules regulating the transfer of water and other compounds such as ions, glycerol, urea, and hydrogen peroxide. Their alteration has been reported in several conditions such as cancer. Tumor progression might be enhanced by aquaporins in modifying tumor angiogenesis, cell volume adaptation, proteases activity, cell–matrix adhesions, actin cytoskeleton, epithelial–mesenchymal transitions, and acting on several signaling pathways facilitating cancer progression. Close connections have also been identified between the aquaporins and hematological malignancies. However, it is difficult to identify a unique action exerted by aquaporins in different hemopathies, and each aquaporin has specific effects that vary according to the class of aquaporin examined and to the different neoplastic cells. However, the expression of aquaporins is altered in cell cultures and in patients with acute and chronic myeloid leukemia, in lymphoproliferative diseases and in multiple myeloma, and the different expression of aquaporins seems to be able to influence the efficacy of treatment and could have a prognostic significance, as greater expression of aquaporins is correlated to improved overall survival in leukemia patients. Finally, we assessed the possibility that modifying the aquaporin expression using aquaporin-targeting regulators, specific monoclonal antibodies, and even aquaporin gene transfer could represent an effective therapy of hematological malignancies.

## 1. General Considerations on Aquaporins

Aquaporins (AQPs) are water channel transmembrane protein molecules that enable water transfer throughout the cellular membrane increasing water penetrability; however, transport rates are also dependent on AQP channel density and subtype-specific single pore properties [1]. These substances include a group of proteins, which are localized in the cell membrane, and 13 different proteins (AQP1–12A, Β) have been identified in humans [1,2,3]. Furthermore, even if the greater part of the AQPs operate easing the water passage, some AQPs, such as AQP3, AQP7, and AQP9, also have a relevant effect on glycerol transport, thus being indicated as aquaglyceroporins [4]. Based on their ability to accelerate the glycerol transport via the plasma cell membrane, the AQPs are further classified into aquaporins, aqua-glycerol-porins and super aquaporins [5,6,7,8,9,10,11,12]. In the same context, AQPs have been correlated with other functions, and some AQPs regulate the transfer of ions, while maintaining their principal task of water transfer [13,14].

**Figure 1:**
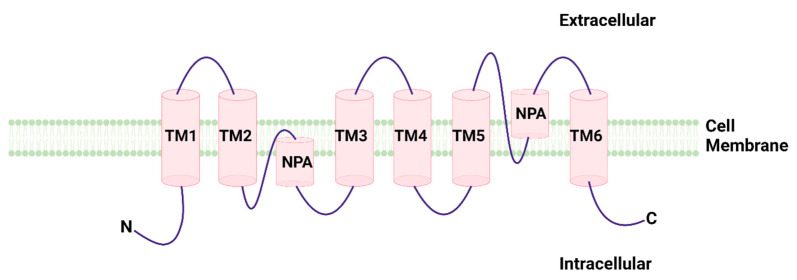
Structural model of aquaporins formed by six transmembrane helices and two helix-forming re-entrant loop containing the signature NPA motif.

Structural analysis on aquaporins have displayed a conserved aquaporin fold where six transmembrane helices contain a small water-directing channel in which waters arrange in a line. A cytoplasmic loop B and an extracellular loop E drop into the membrane from two different positions sides, producing two smaller helices that institute a seventh pseudo-transmembrane portion. In the cell membrane, four aquaporin molecules constitute a homotetramer, thus generating a fifth small channel through its center. This structure is predominantly hydrophobic and is able to support gas transfer. Beside the water-directing channel, two other parts are of special relevance: the NPA-part in the central portion of the channel and the aromatic–arginine (ar/R) part near the extracellular region. The first part is supposed to have a main effect in the proton exclusion system and contains two copies of a Asn-Pro-Ala motif. This area is thought to be the aquaporin signature motif. Nevertheless, several variants are present in the different forms of AQPs, for instance NPC in AQP11, both of which are positioned intracellularly. Furthermore, it has been proposed that the NPA motifs are essential for aiming of aquaporins to the plasma membrane. The ar/R region is composed of arginine and three other amino acids, two of which are conserved in water-specific aquaporins. This part is the tightest point along the channel and operates as a filter, stopping anything greater than water. In aquaglyceroporins, the ar/R region is generally more or less 1 A° wider, allowing transport of bigger compounds [15,16,17] (Figure 1).

AQPs structures determine the pore organization that allows specific, bidirectional transfer of water in some type of AQPs [18], and water and glycerol passage in aquaglyceroporins [19].

Numerous different molecular mechanisms are able to regulate AQPs functions. Modifications in the speed of transcription of AQP genes can cause variations in protein concentrations and modification in membrane permeability. This mechanism is quite longwinded (hours) as after gene transcription, the proteins have to be transferred from the endoplasmic reticulum membrane to the cellular membrane. Modifications in cellular localization of existent proteins can be much quicker. This is due to variations in the speed of internalization via endocytosis and return via recycling endosomes, or by exocytosis. Membrane transferring can cause modifications in the amount of AQP protein in the cell membrane leading to variations in water permeability. Furthermore, other mechanisms might be the onset of variations in the single channel permeability as an effect of post-translational variations, such as protein phosphorylation, or to a protein–protein contact, which can cause modifications in membrane permeability via the so called “gating effect” [20].

AQPs are present in different cells and organs, and based on results provided by experimental animal models, they might have a relevant action in the pathogenesis of acute and chronic pulmonary diseases [21,22,23], and acute renal failure [24].

In addition, AQPs are implicated in cell growth via numerous, different processes, such as modifications in cell volume, perviousness to glycerol and other small substances, and modifying the cell cycle. In fact, situations that can modify cell volume alter cell growth, and increased production of AQP1 or AQP3 provokes a change on the generation of substances which are critical for the progress of the cell cycle [25,26]. On the other hand, AQP3-defective cells displayed a reduction in cell growth, which was correlated to a modified glycerol production, reduced ATP amount, and altered mitogen-activated protein (MAP) kinase signaling pathway [27,28].

**Figure 2:**
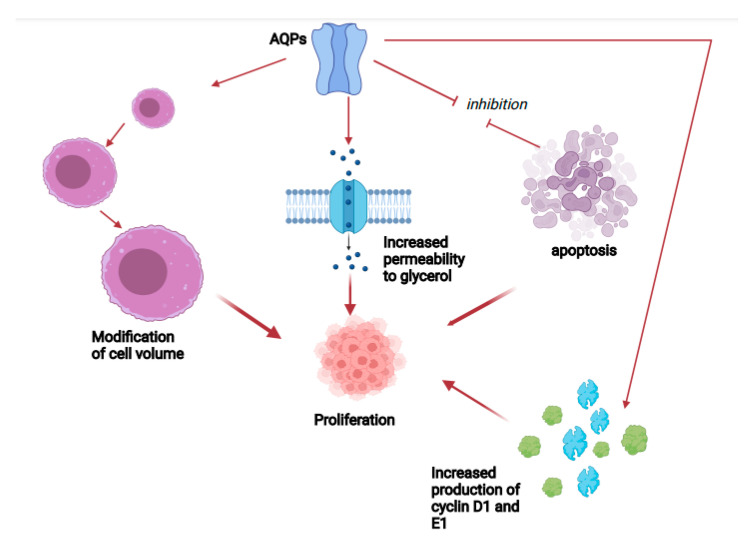
Effects of AQPs on cell proliferation.

A different mechanism might be the onset of resistance to programmed cell death. A study performed employing knockdown of AQP1 displayed in a relevant decrease in the concentration of the antiapoptotic protein Bcl¡2 with a significant increase in the amount of proapoptotic protein Bax and cleaved caspase 3. These findings suggested that the function of AQP1 was most probably due to the stimulation of the G1/S cell cycle transition and to the inhibition of apoptosis [29]. Transfection of PC12 cells, a cell line originated from a pheochromocytoma of the rat adrenal medulla, with AQP1 cDNA enhanced the fraction of cells in S and G2/M phases, and this was correlated to augmented production of cyclin D1 and E1, essential components for cell cycle passage to G1 phase and G1/S transition [30]. This passage from one phase to another is characterized by an increased cell volume, while programmed cell death implicates a decrease in cell size [31,32]. In fact, PC12 cell presenting an enhanced expression of AQP1 show a bigger cellular volume with respect to the wild-type cell; a study confirmed that the change in cellular form provoked by an increased presence of AQP1 accelerates cell growth via the progression of the cell cycle and the reduction in apoptotic dynamics [30] (Figure 2).

## 2. Aquaporins and Cancer

From the above, a role of AQPs in neoplastic pathologies has been proposed. Tumor progression and diffusion might be enhanced by AQPs in modifying cell size control, intermingling with actin cytoskeleton, supporting tumor angiogenesis, modulating cell–cell and cell–matrix contacts, controlling proteases, participating to the government of epithelial–mesenchymal transition (EMT), and managing several signaling pathways [33,34,35,36,37].

The effects of AQPs on angiogenesis could constitute a further step of intervention in neoplastic proliferation as the essential action performed by angiogenic dynamics in the onset and progression of tumors is well known [38,39,40,41,42].

Moreover, AQP3 has been involved in the EMT process as AQP3 increase after epidermal growth factor (EGF) in tumors is concomitant with increased cell motility, diffusion, and metastasis [43].

AQPs could also play a regulatory role on oxidative stress. In fact, in addition to the AQPs classes mentioned above, a further group, peroxiporins, exists which includes paralogs belonging to these groups: AQP 1, 3, 5, 8, 9, and 11. These AQPs have high hydrogen peroxide (H_2_O_2_) permeability and has an effect in ROS scavenging. Some studies assessed a role of AQP6 in driving the H_2_O_2_ efflux from malignant pleural mesothelioma cells, and this action could justify the resistance of this tumor to conventional chemotherapy and radiotherapy [44]. Furthermore, AQP3-mediated H_2_O_2_ transport has been correlated to breast cancer cell migration, and AQP5 facilitating transmembrane H_2_O_2_ diffusion is able to modulate cell proliferation of cancer yeast cells in response to oxidative stress. Cell migration was blocked by AQP3 or AQP5 gene silencing and could be restored by external oxidative stimuli [45]. These findings demonstrate that AQP5 can have a relevant effect on cancer cell; by provoking a fine-regulation of intracellular H2O2, AQP5 is able to stimulate signaling networks correlated to cell growth and survival and to modify cellular resistance to oxidative stress as well as facilitate cancer cell migration, and represents a promising target for the development of tumors treatments. 

Remarkably, AQPs seem able to change the metabolic functions of cells favoring the appearance of tumors [46]. An experiment displayed enhanced AQP1 production after exposure to hypoxia in rat glioblastoma cells; AQP1 expression was correlated with the degree of glycolysis and this finding suggested that AQP1 generation is stimulated by hypoxia-induced glycolysis [47]. Indeed, hypoxia-induced AQP1 generation may happen via the E-Box/ChoRE transcriptional component existent in AQP1 gene promoter, which is recognized as a factor able to increase gene transcription in reaction to enhanced glucose usage and metabolism [47,48,49,50,51,52].

**Figure 3:**
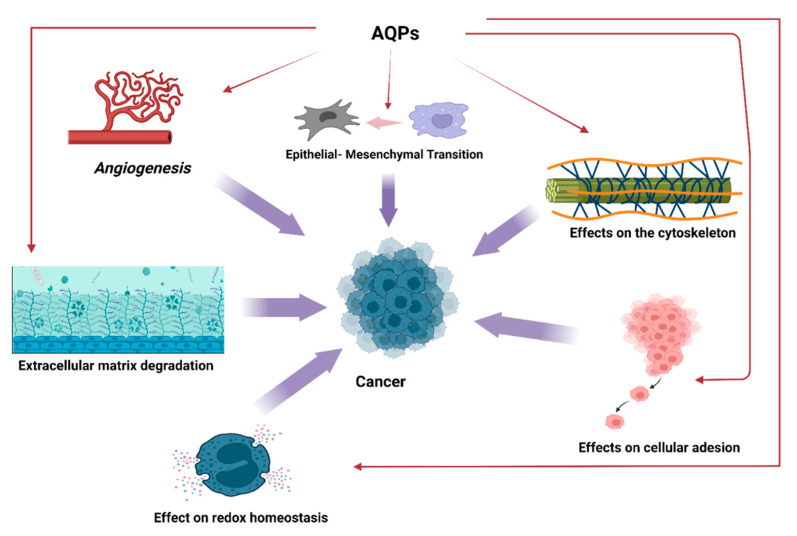
Effects of AQPs in onset and progression of cancer diseases.

Several experiments have then tried to analyze the intimate molecular mechanisms of the effects of AQPs. The ability of tumor stem cells for cell growth and differentiation is due to the action of different signaling pathways [53,54], and AQPs seem to be capable of modulating the effects of many intracellular signals [55,56,57,58,59] (Figure 3).

From a clinical point of view, it was reported that AQP3 and AQP5 are involved in the pathophysiology of esophageal, gastric, pancreatic, hepatocellular, colorectal, breast and cervical cancers [60,61,62,63,64,65,66,67,68,69]. In a recent metanalysis, increased production of AQP1and AQP5 was correlated with reduced survival rate, bad prognosis, and lymph node diffusion; remarkably, tumor chemotherapy caused AQP decrease [70,71,72,73].

An increased generation of AQP3 was also correlated with severity of metaplasia, bad prognosis, augmented self-renewal, and cancer diffusion, while a decreased generation of AQP3 was correlated to defective growth of tumor cells, reduced proliferation and decreased motility and diffusion of tumor cells [72,73].

The studies on the role played by AQPs in the onset and progression of hematological neoplasms appear less numerous in the literature. The aim of this review was to analyze the molecular mechanisms that regulate cell proliferation in these pathologies and to evaluate the possibility that interfering with the expression of AQPs could constitute a valid and safe treatment option.

## 3. AQPs and Hematological Malignancies

Close connections have been identified between the AQPs system and hematopoietic cells. In fact, it is interesting to note that AQPs could play an important role not only in hematological neoplasms, but also in the normal hematopoiesis process. Stimulating studies have analyzed the effect of AQPs in the erythrocyte differentiation using erythroleukemia cells [74]. A study has displayed that in erythroleukemia HEL and K562(S) cells, the AQP1 transcript expression was stimulated by sodium butyrate, which is a powerful factor for erythroid differentiation. In addition, a putative butyrate-response component has been recognized in the promoter sequence of the AQP1 gene. After butyrate-provoked erythroid differentiation, AQP1 transcript production increased significantly, inducing the occurrence of water-permeable cells with presence of AQP1 on plasma cell membrane. In addition, a K562(S) displayed high butyrate-caused expression of functional AQP1, while AQP1 promoter activity was much higher in HEL and K562(S) cells than in nonerythroid cells, suggesting the existence of erythroid-specific elements [75].

In a different animal erythroleukemia cell line (MEL cells), the AQP1 protein expression was provoked by dimethyl sulfoxide and corticosteroid [76]. These data were confirmed by the same group of researchers and could also be important in the framework of erythroleukemia itself. In fact, retinoic acid (RA) could cause differentiation of erythroleukemia cells in the direction of red blood cells [77], and AQP1 synthesis is particularly stimulated by RA in HEL cells; both all-trans-RA (ATRA) and 9-cis-RA (9CRA) intensely stimulated the expression of AQP1 mRNA and protein in a dosage-dependent modality. AQP1 was mainly present in plasma cell membrane in cells treated with RA. Data suggested that RAs intensely stimulated the AQP1 gene expression via the RA response element and delineate a new role in the control of erythropoiesis [78].

However, AQPs may also have an effect in different types of acute myeloid leukemia (AML), a severe hematologic neoplasia affecting older patients. It is characterized by an intense clonal proliferation of tumoral myeloid stem cells in the bone marrow, due to the onset of malignant alteration of hematopoietic stem cells that go through succeeding genetic changes, finally giving rise to an overt malignancy. Biological features of AML are enormously composite with relevant genetic, epigenetic, and phenotypic variants, which differ from each other by molecular and genomic modifications involving genes that are implicated in cell growth, differentiation and survival [79,80].

Arsenic trioxide (ATO) is extremely effective for the treatment of acute promyelocytic leukemia (APL), but it is unable to produce adequate results in other types of AML patients with non-APL diseases. In fact, the greater part of non-APL AML cells presents small concentrations of the ATO transporter AQP9 protein, making them less responsive to ATO administration. Lately, an experiment showed that granulocyte-colony stimulating factor (G-CSF) can increase the expression of AQP9 [81]. The authors supposed that the administration of G-CSF may foster the antileukemic action of ATO in non-APL AML cells. In the report, non-APL AML cell lines including HL-60 and THP-1 were treated with G-CSF, and then with ATO. The combined use of G-CSF with ATO provoked programmed cell death more intensely with respect to ATO alone. G-CSF increased the expression of AQP9 and increased the intracellular levels of ATO in AML cells. When AQP9 expression was enhanced, it significantly boosted the cytotoxic effect of ATO. Conversely, when AQP9 was reduced, it markedly lowered the cytotoxic action. Moreover, an experiment demonstrated that the increase in AQP9 by G-CSF is due to the transcription factor CCAAT enhancer binding protein beta (CEBPB). Interestingly, the study reported that the combined use of G-CSF and ATO remarkably reduced tumor proliferation in a xenograft animal model [81]. Therefore, the combined employment of G-CSF and ATO increasing AQP9 concentrations would be a possible therapeutic option for AML subjects. Furthermore, a study confirmed that the expression of AQP9 was greater in NB4 cells than in THP-1 and HL-60 cells [78]. This may partially clarify the reason why ATO is more efficient in APL than nonM3 AML. Indeed, the amount of AQP9 mRNA, as evaluated by Q-PCR, was inversely correlated to cell survival after treatment in 2 μM As_2_O_3_ for 48 h [82].

The relationship between the concentrations of AQP9 and response to ATO was verified in other experiments employing two APL cell lines, HT93A and NB4, and primary APL cells from APL patients [83]. A greater response to ATO-stimulated programmed cell death was reported in the NB4 cells with respect to that in HT93A cells. Furthermore, in NB4 cells, the expression of AQP9 mRNA was reported at a small but measurable concentration at cycle 33, and increased rapidly with the amplification. Conversely, only a measurable concentration of AQP9 was reported in the HT93A cells at cycle 39, suggesting much greater expression quantities of AQP9 mRNA in NB4 cells with respect to HT93A cells. Moreover, the expression concentrations of AQP9 protein in NB4 and HT93A cells were evaluated employing flow cytometry, and according with the expression level of AQP9 mRNA, a much greater expression level of its protein was also reported in the NB4 cells with respect to the amount in the HT93A cells. Finally, similarly to APL cell lines, the expression of AQP9 was strongly associated with the ATO-mediated stimulation of programmed cell death in primary APL cells. Contrariwise, no relationship was reported between ATO response correlated with AQP9 expression amounts and chromosomal alterations [83]. These findings suggest that the concentrations of AQP9, more than other different biomarkers, related with the response to ATO in APL cell lines and primary cells from APL patients. These results also indicate that the AQP9 generation in APL subjects is a prognostic biomarker for the positive effect of ATO administration, as AQP9 has a fundamental relevance in multiple arsenite-mediated action on normal and leukemic cells.

A different mechanism by which AQPs may intervene on ATO and ATRA actions on leukemic cells, is related to isomerase Pin1, a controller of oncogenic signaling, which is activated in several tumors and is able to stimulate about 43 oncoproteins, disables more than 20 tumor suppressors, operating as a post-phosphorylation controller of oncogenic networks [84,85,86,87,88]. Kozono et al. evaluated AQP9 and its relationship with Pin1. AQP9 was identified in ATO-responsive cells, but not in ATO-resistant cells, and AQP9 presence was inversely related with Pin1 concentration and cell proliferation. ATO’s capability to block tumor proliferation was positively related with Pin1 degradation and AQP9 presence and Pin1 degradation. To explain the functional impact of AQP9 in modifying ATO sensitivity, they inhibited AQP9 expression in ATO-sensitive cells and increased AQP9 in ATO-resistant cells. Silencing AQP9 annulled the capability of ATO to cause Pin1 degradation and blocked cell proliferation. Contrariwise, AQP9 enhanced expression transformed ATO-resistant cells into ATO-sensitive cells in terms of proliferation reduction and Pin1 degradation. These findings are also confirmed by evaluating cellular ATO uptake as AQP9 inhibition decreased ATO absorption in ATO-sensitive cells, while increased AQP9 expression fostered ATO uptake in ATO-resistant cells. So, ATO absorption through AQP9 controls the capability to provoke Pin1 degradation and block tumor proliferation [89].

This hypothesis was confirmed in a different study in which AQP9 generation was analyzed in ATRA-administered HT93A cells. After employment of ATRA for 7 days, ATRA enhanced AQP9 expression in HT93A cells. ATO also increased AQP9 expression, but the production was smaller than that reported after ATRA [90] (Table 1).

Other molecular mechanisms have been recognized as essential moments of the effect exerted by AQPs on leukemic cells, such as their ability to modify the redox homeostasis. Reactive oxygen species (ROS) can work as regulators in cell signaling systems, and it is ascertained that an increase in intracellular ROS concentration causes cell proliferation, participating to tumor progression [91,92,93]. Amongst ROS, H_2_O_2_ is considered the principal effector in redox signaling owing to its steadiness and capability of diffusion [94].

H_2_O_2_ has long been supposed to penetrate through cell membranes by simple diffusion, but new findings revealed an effect of AQPs in determining H_2_O_2_ transfer throughout the biological membranes [95]. In fact, it has been reported that AQP1 is capable to allow the passage of H_2_O_2_ in a rat smooth muscle cell line modulating redox signaling pathways. It was also reported that AQP3 and AQP8 can regulate H_2_O_2_ intracellular concentrations and are involved in H_2_O_2_-controlled signaling in different cell lines [96]. Interestingly, the AQP types able to allow the passage of H_2_O_2_ are also extensively present in blood cells; AQP1 was reported in the erythrocytes, where it is one of the most copious proteins [97,98,99,100].

An experiment studied if different AQPs can ease the transfer of Nox-derived H_2_O_2_ throughout the cell membrane of leukemia cells modifying several pathways correlated to cell growth. In this work, authors demonstrated that AQP block provoked a reduction in intracellular ROS store in leukemia cells both when H_2_O_2_ was generated by Nox enzymes and when it was supplemented. Furthermore, an AQP8 increase or reduction provoked a different ability of VEGF of causing an H_2_O_2_ intracellular level increase or reduction [101]. Finally, the report displayed that AQP8 is capable of enhancing H_2_O_2_-caused phosphorylation of PI3K and p38 MAPK and that AQP8 generation modified positively cell growth.

A different study performed by the same group of researchers clarified the effect of AQP8 in the redox balance in human leukemia B1647 cells that constitutively generate VEGF [102]. AQP8 overexpression or silencing caused a different effect on the modulation of VEGF capability of increasing or reducing, respectively, H_2_O_2_ intracellular concentration. Moreover, results obtained by a dimedone-based immunochemical technique for sulfenic acid identification revealed that the generation of AQP8 can regulate the action of redox-sensitive targets. AQP8 modified VEGF-provoked redox signaling by promoting the sulfenation of the tumor suppressor PTEN, causing its deactivation and provoking Akt stimulation [102]. Therefore, this technique allowed the researchers to clarify the effect of AQP8 on the delicate regulation of cysteine oxidation in target proteins involved in leukemia cell growth pathways.

However, the actions operated by AQPs on redox systems seem to be absolutely specific, differing according to the AQP studied. NAD(P)H oxidases seem to be stimulated within distinct subcellular spaces, and this placement eases a spatially restricted ROS generation, which, retaining redox-responsive targets in nearness, may allow ROS to stimulate specific redox signaling effects [103]. As it has been reported in different experimental models that lipid raft domains include both Nox enzymes and AQP8 [104], it is possible to conjecture that membrane location has an effect in justifying different behaviors and comportment of AQP3 and AQP8 in stimulating redox pathways even if both AQPs can theoretically ease H_2_O_2_ absorption.

However, the most significant actions exerted by AQPs in the framework of leukemic pathologies remain those on signaling pathways, which might also give reason for the prognostic role in AML patients. An investigation showed that AQP1 works as a tumor suppressor gene and reduces Wnt signaling by acting with GSK3b, b-catenin, LRP6, and Axin1 [105].

As far the prognostic value of AQPs, frequent mutated genes in AML subjects include WT1, FLT3-ITD, NPM1, and CEBPA, which are related to different prognostic impact. Although new techniques, such as next-generation sequencing, have been employed in defining risk definition in young and middle-aged leukemic subjects [106], in elderly AML patients, prognostic stratification is more complex. A different experiment evaluated the prognostic significance of AQP-1 in aged cytogenetically normal AML (CN-AML) and elaborated a new scoring based on AQP1 methylation [107]. In the first and second prognostic group, AQP1 displayed reduced expression in CN-AML with respect to controls, whereas greater expression of AQP1 and AQP1 promoter hypomethylation were correlated to improved overall survival (OS). To understand the fundamental mechanisms, the authors analyzed differentially expressed genes (DEGs) correlated with AQP1 methylation. They established a three-gene prognostic profile founded on AQP1 methylation, which was strongly associated with OS. Thus, AQP1 methylation could be employed as a prognostic marker in aged CN-AML [107].

These results have been confirmed by other studies. A report showed that an increased expression and hypomethylation of AQP1 was correlated with better OS in aged CN-AML. Furthermore, to analyze the significance of AQP1 methylation in the outcome of aged CN-AML, they performed a multi-omics investigation evaluating AQP1 DNA methylation of non-coding genetic material methylation loci, and several cell signaling pathways. Notably, they recognized an AQP1-particular methylated site cg09676669 as a possible diagnostic and prognostic marker for aged CN-AML subjects. Thus, by evaluating the methylation amount of this specific site, it is possible to calculate the outcome of aged CN-AML subjects [108].

These considerations have paved the way for a possible therapeutic use of the modulation of AQPs. It is well known that active passage of ions through cell membrane will be stimulated by osmotic shocks to restore the imposed volumetric change [109,110], and it is conceivable that tumor and healthy cells may respond differently to the same volume changing cue. It is well-documented that active cross membrane transport of ions will be triggered by osmotic shocks to restore (or delay) the imposed volumetric change [109,110], and it is conceivable that tumor and healthy cells may respond distinctly to the same volume changing cue.

In a study, Hui et al. elaborated a system in which specific modifications in the cellular size can be opportunely caused by changing the voltage put across a Nafion membrane that divides the culture medium from a reservoir [111]. It was reported that active ion transfer through the membrane of leukemia K562 cells will not be stimulated by a slow modification in the extracellular osmolarity. Moreover, when exposed to the identical voltage, tumor cells will have a 5–10% greater death percentage with respect to normal cells. The authors demonstrated that this effect is essentially provoked by the increase in AQPs4 expression in neoplastic cells, while knockout AQP4 cells presented a strongly decreased modification in the cellular size and cell death [111].

Several compounds of natural origin appear to be also able to modify the production of AQPs. Among cruciferous vegetables, broccoli holds the greatest level of the glucosinolate glucoraphanin, which is metabolized by myrosinase, producing sulforaphane (SFN), a substance which has an antitumoral effect, and neuroprotective and anti-inflammatory properties, indicating a multiple protective effect [112]. The powerful antitumor action of SFN is correlated with its capability to influence several different systems which regulate tumorigenesis. Numerous studies displayed that SFN inhibit tumor onset by blocking phase I enzymes [113] and stimulating phase II detoxifying enzymes [114]. Furthermore, SFN inhibits tumor cell growth via the regulation of genes implicated in programmed cell death, cell cycle arrest, cancer diffusion, and angiogenesis [115,116,117,118,119].

However, SFN cytotoxic actions have also been reported in hematological malignancies [120], and it has been demonstrated that SFN exposure of HL-60 cell line and acute lymphoblastic leukemia cells stimulated programmed cell death [121,122,123,124]. Prata et al. assessed if SFN could modify AQP8-induced H_2_O_2_ transfer and NADPH oxidase (Nox)-induced H_2_O_2_ generation in B1647 cells, a cell line originated from a subject with AML with a complete erythro-megakaryocytic phenotype [125]. In order to confirm this hypothesis, B1647 cells were exposed to different SFN levels for 24 h, and the expression of AQP8 was analyzed by RT-PCR and Western blot techniques. Data suggested that AQP8 was remarkably reduced both at transcriptional and protein level upon cell treatment with 10 μM SFN, whereas 1 or 5 μM SFN did not cause any significant change. Furthermore, the results obtained by coimmunoprecipitation method suggested that these two molecules are strictly correlated to each other. Cell exposure to SFN also decreased the production of peroxiredoxin-1, which is increased in practically all AML forms. Remarkably, SFN levels capable of provoking these results are reachable by a normal dietary ingestion of cruciferous vegetables.

The effect exerted by AQPs on myeloid cell proliferation does not seem to be limited to acute forms of leukemia but is also expressed in chronic forms. Chronic myeloid leukemia (CML) is a hematological tumors distinguished by the clonal growth of white blood cells that are derived from the myeloid line in the bone marrow. CML is due to the t(9;22)(q34;q11) reciprocal translocation between chromosome 9 and 22 that generates the Philadelphia chromosome. This phenomenon produces the fusion of the Breakpoint Cluster Region (BCR) gene with the Abelson proto-oncogene 1 (ABL1) gene, causing the BCR::ABL1 fusion gene. The resultant chimeric protein, BCR::ABL1, is a powerful tyrosine-kinase signaling protein that increases cell growth and decreases programmed cell death, which in turn provokes the onset of leukemia [126].

Chae et al. evaluated the AQP5 generation profile and its effect in chronic myelogenous leukemia (CML) [127]. The authors evaluated the expression of AQP5 in CML cells. While normal bone marrow biopsy samples displayed no expression of AQP5, one third of CML samples displayed AQP5 expression. Moreover, AQP5 expression level increased with the occurrence of imatinib mesylate resistance. The authors reported that the enhanced expression of AQP5 in K562 cells caused increased cell growth, while small interfering RNA aiming AQP5 decreased cell growth in both K562 and LAMA84 CML cells. Finally, by immunoblotting and flow cytometry, they demonstrated that phosphorylation of BCR-ABL1 is fostered in AQP5-overexpressing CML cells and reduced in AQP5 siRNA-treated CML cells. Remarkably, caspase 9 activity increased in AQP5 siRNA-treated cells, while FISH demonstrated no evidence of AQP5 gene amplification in CML from bone marrow.

These results may offer the starting point for a new CML treatment aiming at AQP5.

## 4. AQPs and Lymphoproliferative Diseases

Lymphomas are a heterogenous set of hematologic malignancies originated from B cells, T cells or natural killer (NK) cells that generally arise in the lymph nodes or in different lymphoid structures, but can also happen in numerous other organs. The last classification of lymphoid tumors identified more than 80 lymphoma forms which vary from each other as for cytological and histopathological features, immunophenotype, and genomic alterations. Moreover, lymphomas show a significant biological heterogeneity, which is mirrored in an equally marked clinical heterogeneity, varying from highly aggressive forms to indolent ones [128].

AQPs seem to be able to intervene also on the proliferation of lymphoid-type neoplastic cells. Chronic lymphocytic leukemia (CLL) is a tumoral proliferative condition generally involving mature B lymphocytes [129,130] and is the most common adult leukemia in the Western world.

Nuclear Factor of Activated T cells 5 (NFAT5) is the main stimulated transcription factor after osmotic pressure increase, and it also controls several genes able to modify many cellular activities. The consequences of NFAT5 on neoplastic proliferation have been extensively evaluated. A report revealed the presence of enhanced production of NFAT5 in CLL subjects. In addition, NFAT5 reduction decreased cell growth and promoted programmed cell death of CLL cells. Other studies displayed NFAT5 controlled AQP5 expression and the phosphorylation of p38 MAPK. The authors also assessed that AQP5 overexpression increase abolished the blocking action of NFAT5 reduction on cell growth in CLL cells. They also demonstrated STUB1 connected to NFAT5 and stimulated its destruction. These data suggest the participation of AQP5 and NFAT5 in CLL diffusion and propose that AQP5 and NFAT5 could operate as a hopeful therapeutic target for CLL therapy [131].

Still in the framework of chronic lymphoproliferative diseases, an investigation assessed the correlations between AQPs production and the features of blood vessels in patients affected by diffuse large B-cell primary central nervous system lymphomas [132]. Data displayed that a sustained AQP4 expression was associated with an elevated Ki-67 index and aAQP4 marked tumor and endothelial cells in cytoplasm and plasma membranes. AQP4 expression was reduced in lymphoma areas with a decreased Ki-67 index where rare tumor cells were positive to AQP4, and endothelial cells displayed AQP4 expression on their abluminal side [132]. In general, these results indicate the relevance of AQP4 in primary central nervous system lymphomas. This AQP might be involved both in cerebral oedema development and resolution and in lymphoma cell diffusion activity.

An involvement of AQPs has been also reported in primary effusion lymphoma (PEL), an aggressive AIDS-linked KSHV-associated non-Hodgkin’s lymphoma, which is characterized by the increased production of AQP3 [133]. Employing nimesulide, a notorious COX-2 specific non-steroid anti-inflammatory drug, a different study reported that nimesulide is effective in provoking a reduction in cell growth in PEL, and Burkitt’s lymphoma. Nimesulide altered p53-LANA-1 protein complexes and stimulated the p53/p21 tumor-suppressor system. COX-2 inhibition reduced cell survival kinases and PEL-defining genes such as AQP3, causing cell death and G1 block [134]. An experiment assessed that the AQP3 gene polymorphism is correlated with an augmented occurrence of Epstein–Barr virus (EBV)-associated lymphoma (EBVaL), and the homozygous AA genotype is more commonly reported in subjects who have EBVaL [135].

Finally, AQPs appear to have an effect also in the framework of monoclonal gammopathies by interfering in myelomagenesis. Multiple myeloma (MM) is a tumor of plasma cells (PCs) that growth and collect within the bone marrow (BM), provoking anemia, renal failure, hypercalcemia, and lytic lesions. It represents 10–15% of all hematological malignancies, with a global prevalence of about 160,000 new diagnoses and 100,000 deaths per year, and a 5-year survival percentage around 50%. PCs are generally postmitotic cells, derived from B cells within the germinal center. PCs produce immunoglobulin and are contained in the bone marrow, spleen, and gut BM [136].

Experimental data displayed that bone marrow samples of subjects with active MM evidence remarkably greater concentrations of AQP1 with respect to those from subjects with non-active MM, whose concentrations are increased, but to a lower level, than those of subjects affected by monoclonal gammopathies of undetermined significance (MGUS). MGUS patients presented the same AQP1 expression than those of subjects affected by anemia due to vitamin B12 or iron insufficiencies. Immunohistochemistry evaluation of AQP1 highlighted bone marrow microvessels whose density was remarkably higher in MM subjects with active disease and always strictly associated with the microvessel area when evaluated with factor VIII-related antigen/von Willebrand’s factor (FVIII-VWF). Thus, the data indicated that among plasma cell malignancies, AQP1 expression is specially correlated with microvessels of MM and that the greatest grade of expression happens in active MM with increased angiogenesis, in which AQP1 identifies less mature neovessels than FVIII-VWF [137]. It may, possibly, enhance angiogenesis in positive feedback, and thus, it may enhance MM progression. This fact must be evaluated for therapeutic vascular targeting.

In fact, the effects exerted by AQPs in MM could be employed for the treatment of the disease. As reported above, ATO is a compound used for APL therapy. However, several experiments have reported that ATO can be employed to cure MM, although the response of MM to ATO is dosage dependent, and the therapeutical results of small dosages are inadequate [138]. As said in a previous section, several findings indicated that neoplastic cell response to ATO is correlated to the intracellular arsenic concentration, and AQP9 is the main element that manages intracellular arsenic content. Curcumin is a secure and efficacious substance that can increase the antitumor activity of several drugs [139], and a study assessed if curcumin could enhance ATO effects in MM cells and if this effect is correlated to the control of intracellular arsenic store [140]. U266 cells were exposed to curcumin, ATO, curcumin, and the authors evaluated MM cell growth, programmed cell death, and intracellular arsenic amount. ATO-caused death of U266 cells happened in a dosage-dependent modality, and the effects at small doses were limited. Curcumin remarkably increased the cytotoxicity of ATO against U266 cells. The arsenic intracellular amount in U266 cells in the group treated with both drugs increased with respect to ATO treatment alone. Remarkably, the AQP9 mRNA protein expression also augmented in U266 cells [140]. Thus, curcumin can increase the cytotoxic action of ATO on MM cells by enhancing the intracellular arsenic amount, which may be correlated to the increase in AQP9 expression.

## 5. Future Perspectives

Water represents about 50–60% of body mass, and control of water balance is essential for all existing organisms; AQPs have a main effect in conserving ideal equilibrium and their modification is capable of provoking relevant diseases [141].

However, even though the dimension and structure of AQP pores are recognized up to atomic detail, no clinically beneficial pore-inhibiting substances for any AQP have until now been identified, and their employment is essentially restricted to in vitro or preclinical experiments [142]. Furthermore, procedural dissimilarities in implementing investigations may also cause misleading results, and the real efficacy of several AQP controllers is thus debatable [143].

Despite this, some experimental results appear particularly encouraging, and there is the possibility to employ AQP-targeting treatments, such as AQP-targeting blockers, AQP-specific monoclonal antibodies, and even AQP gene transfer is real [144].

For instance, AER-270 is a specific AQP4 blocker, and AER-271, a pro-drug of AER-270, has been employed in a phase I experiment performed in normal subjects (NCT03804476). However, only 20% of block of human AQP4 was described, with respect to 70% maximal block of rat AQP4 [145].

As mentioned in the previous sections, it was found that curcumin, the functioning component of the spice turmeric (*Curcuma longa Linn*), decreased AQP3 production and diminished cell motility in tumor cells, possibly by its inhibitory action exerted on EGFR and the protein kinase B/ERK stimulation [146]. In the same way, still in the framework of natural compounds, realgar-Indigo naturalis formula (RIF) has been reported to be very efficacious in treating APL. The main elements of RIF are realgar, *Salvia miltiorrhiza*, and *Indigo naturalis*, with tetra-arsenic tetra-sulfide and they can increase the production of AQP9 and ease the transportation of tetra-arsenic tetra-sulfide into APL cells, which increases their therapeutic effectiveness [147]. Contrariwise, *Bacopa monnieri* is a plant originated from India that is employed in alternative medicinal treatments. The main components are bacopaside-I and bacopaside-II, which can block AQP1 but not AQP4 activities [148].

Several other molecules seem to be able to control the production and regulate the effect5 of AQPS [149]. Acetazolamide is theoretically suitable as an angiogenic blocker by controlling AQP1 activity in different tumor experimental models, and it can also inhibit xenograft cancer proliferation in nude mice, partially repressing the AQP1 gene expression [150].

A different blocker is the tetraethylammonium which is a reversible blocker of AQP1, AQP2, and AQP4 [151]. Conversely, estradiol is a hormone which is able to enhance AQP2 production and considerably fostered the motility, diffusion, growth, and adhesion of Ishikawa cells (endometrial adenocarcinoma). The employment of the estrogen receptor inhibitor ICI 182780 was capable of decreasing this action on AQP2 and tumor cells [152].

Furthermore, although Bumetanide demonstrated a small inhibiting action on AQP4, employing bumetanide as an initial support, some authors developed a new synthetic substance, AqB013, and displayed that it was an efficacious blocker of AQP1 and AQP4 [153]. AqB011 provoked a dosage-dependent blockade of the AQP1 ion pore but did not exert any effect on water transfer [154,155,156]. AqB011 showed encouraging results as a potential add-on therapy for reducing tumor diffusion, by inhibiting the AQP1 ionic conductance that eases tumor motility in AQP1-positive tumors [157].

As reported above, the expression concentration of AQP5 was rather greater amongst subjects with CML who acquired resistance to imatinib mesylate. Yang et al. displayed that AQP5 protein had a positive correlation with cell proliferation, and the production of AQP5 could be reduced by cisplatin administration [158].

Different blockers of AQPs are obtained from metals, and several AQPs are blocked by sulfhydryl-reactive mercurials such as gold and mercury [159], but these elements are totally non-specific in their effects and extremely toxic. For instance, auphen is a gold-originated complex which, when employed at dosages of 100 µM, totally inhibits AQP3-derived glycerol transfer and water transfer by 20% in human erythrocytes [35]. It also reduces growth in several cell lines by blocking AQP3 glycerol transfer [36].

Other possible inhibitors of AQPs that are heavy metals have also been identified, including zinc, and copper chelator ATN-22484 [160,161].

Further supposed putative small molecule AQP blockers have come into view from computational analyses, with several AQP1 inhibitors, and one AQP1 stimulator. Substances #1, #2, and #3 were recognized by computational evaluation and examining their ability to inhibit the osmotic enlarging in AQP1-presenting Xenopus oocytes [162]. In fact, these compounds decreased osmotic swelling by about 80%, but were not able to block AQP1 in red blood cells. Compound #4 (AqB013), a substance similar to the NKCC1 inhibitor bumetanide was recognized to block both AQP1 and AQP4 [153]. However, this compound did not exhibit in vivo the expected effects when examined in a brain damage experimental model [163]. The same group also assessed that an analog of the furosemide, AqF026, stimulated AQP1 by about 20% in the oocytes [164]. Other substances were stated to block AQP [165], but their configurations are not drug-like, and they are probably toxic compounds. More recently, other compounds were identified but their activities were extremely irregular [166,167].

Different experimental approaches have been performed to discover molecules capable of controlling the effect of AQPs. Although the detection of a neutralizing anti-AQP antibody is improbable due to its great molecular dimensions and connecting to extracellular loop places far from the AQP pore, AQP-joining antibodies have been experimented with, albeit with different applications than cancer treatment. In one experiment, Tradtrantip et al. produced an anti-AQP4 antibody (“aquaporumab”) in which the antibody Fc portion was changed to operate on complement- and cell-mediated cytotoxicity [168]. This antibody was able to prevent in vitro cytotoxicity from neuromyelitis optica patient sera and avoided demyelination in experimental animal models. Other studies of small molecule blockers of AQP4-IgG binding to AQP4 selected different compounds, but their affinities were too small to be employed as drugs [169].

Furthermore, generally, the effects of the analyzed AQP inhibitors have not been validated on retesting. This is probably due to methodological problems in water transfer, and to the difficulties in controlling the effects on the pore-containing membrane proteins. Other problems include the broad allocation of AQPs, the structural similarity of different AQPS, and the ability of water to bypass barriers [170].

Finally, AQP gene transfer has been also evaluated for clinical treatment of non-hematological patients for head or neck tumors. Patients were treated with AQP1-cDNA transfer treatment in a Phase I clinical trial [171]. However, the efficacy and security of this therapy require more investigation.

## 6. Conclusions

Clinical and preclinical studies highlighted that AQP expression is modified in a number of solid and hematological tumors. Thus, in recent years, biological activities and signaling pathways of AQPs in neoplastic diseases have been assessed in a condition of AQP modification, employing genetic strategies.

However, though AQPs are proved drug targets, it is uncertain if their blockade or regulation is capable of causing important clinical effects on hematological patients. Elaboration of reliable tests to evaluate and validate possible AQP regulators across various in vitro and in vivo experimental models will clarify the effective potential of these substances. In any case, pharmacological regulation of AQP activity is considered as an encouraging approach for bettering hematological malignancy therapy, especially in combined administration with other drugs that could synergistically influence the processes of angiogenesis, growth, and programmed cell death that sustain tumor progression.

## Figures and Tables

**Table 1:** Involvement of AQP9 in the response to drugs employed for the treatment of acute myeloid leukemia.

Disease	Drug	Study	AQP	Effect	Mechanism	Ref.
AML	G-CSF + ATO	In vitro (HL-60, THP-1)	AQP9	Apoptosis	Increased intracellular ATO	[81]
AML	GSF + ATO	In vivo xenograft animal model	AQP9	Reduced tumor proliferation	CEBPB	[81]
AML (APL)	ATO	In vitro (HT93A, NB4)	AQP9	Apoptosis		[83]
AML	ATO + ATRA	In vitro	AQP9	Reduced pro-oncogene effect	Effect on Pin1	[89]
AML	ATO and/or ATRA and/or G-CSF	In vitro (HT93A)	AQP9	Decreased cell viability	Increased arsenic uptake	[90]

Acute myeloid leukemia (AML); acute promyelocytic leukemia (APL); arsenic trioxide (ATO); all-trans retinoic acid (ATRA); Granulocyte colony-stimulating factor (G-CSF); CCAAT enhancer binding protein beta (CEBPB); Protein interacting with never in mitosis A1 (Pin1).
